# M2 macrophage-mediated interleukin-4 signalling induces myofibroblast phenotype during the progression of benign prostatic hyperplasia

**DOI:** 10.1038/s41419-018-0744-1

**Published:** 2018-07-09

**Authors:** Jindong Sheng, Yang Yang, Yun Cui, Shiming He, Lu Wang, Libo Liu, Qun He, Tianjing Lv, Wenke Han, Wei Yu, Shuai Hu, Jie Jin

**Affiliations:** 1 Department of Urology, National Research Center for Genitourinary Oncology, Peking University First Hospital, 8 Xishiku Street, Xicheng District, Beijing 100034, China; 2 Department of Urology, Beijing Chaoyang Hospital, Capital Medical University, Gongti South Road, Chaoyang District, Beijing 100020, China

## Abstract

Benign prostatic hyperplasia (BPH) is a progressive disease in elderly men, but potential factors accelerating its progression remain largely unknown. The aim of this study was to elucidate the factors affecting BPH progression by understanding the complex mechanisms causing early- progressed BPH, which progresses rapidly and requires surgical intervention before the age of 50. Three groups of human prostate tissue samples, from patients with early-progressed BPH, age-matched prostate and elderly BPH tissues, were collected (*n* = 25 each). We compared these tissues to determine the histologic features and molecular mechanisms underlying BPH progression. We found that early-progressed BPH samples were characterised by aberrant stromal hyper-proliferation, collagen deposition and increased M2 macrophage infiltration, compared to those from age-matched prostate and elderly BPH tissues. The M2 macrophage–fibroblast co-culture system demonstrated that the myofibroblast phenotypes were strongly induced only in fibroblasts from the early-progressed BPH samples, while the co-cultured M2 macrophages expressed high levels of pro-fibrotic cytokines, such as IL4 and TGFβ1. M2 macrophage-derived IL4, but not TGFβ1, selectively induced the myofibroblast phenotype through the JAK/STAT6, PI3K/AKT and MAPK/ERK signalling pathways in the early-progressed BPH prostate fibroblasts. Taken together, our results indicate that induction of the myofibroblast phenotype may lead to BPH progression through M2 macrophage-mediated IL4 signalling, and that IL4 may represent a potential therapeutic target, allowing the prevention of M2 macrophage activation and fibroblast-to-myofibroblast differentiation.

## Introduction

Benign prostatic hyperplasia (BPH) is the most frequently diagnosed urological disease in elderly men^1^. The prevalence of BPH is age dependent, with an initial development usually after 40 years of age; the incidence rates are ~50% and 90% in 60- and 90-year olds, respectively^2,3^. Aetiologically, BPH is characterised by an excessive proliferation of both epithelial and stromal cells within the transitional zone, leading to prostate enlargement and lower urinary-tract symptoms (LUTS)^4^. Furthermore, BPH is a chronic progressive disorder^5^, the clinical progression of which is characterised by deterioration of the LUTS, occurrence of acute urinary retention or the need for surgical treatment^6^. In clinical practice, however, only 8% of patients with BPH, show rapid disease progression and require surgical intervention before 50 years of age^7^. In this study, we defined such patients as early-progressed BPH cases, representing a model of rapid BPH progression.

Multiple risk factors, including ageing, hormonal alterations, increased sympathetic nerve activity and chronic inflammation have been proposed to be involved in the pathogenesis and progression of BPH^8^, but no consensus on the main cause of this disease has been reached. The majority of aetiological postulates indicated that chronic histological inflammation could be an originator and facilitator of BPH^9^. Indeed, almost all prostate tissues from patients with BPH showed increased infiltration of immune cells, as well as enrichment of inflammatory mediators and growth factors in the tissue environment^10^. Subsequent inflammatory tissue damage and chronic process of repetitive wound healing and tissue remodelling could lead to a prominent proliferation of stromal and epithelial cells, facilitating BPH progression^9^.

Given that early-progressed BPH is a manifestation of BPH progression, better understanding of the complex mechanisms causing it might help elucidate the causes of BPH progression. Therefore, in this study, we conducted a comparative analysis of three groups of prostate tissues, obtained from patients with early-progressed BPH, age-matched patients with bladder cancer who underwent radical cystectomy and prostatectomy and from elderly patients with BPH. The histologic features and molecular mechanisms leading to the rapid progression of BPH were explored.

## Results

### Early-progressed BPH is a fibrosis-associated disease with excessive collagen deposition

**Fig. 1:**
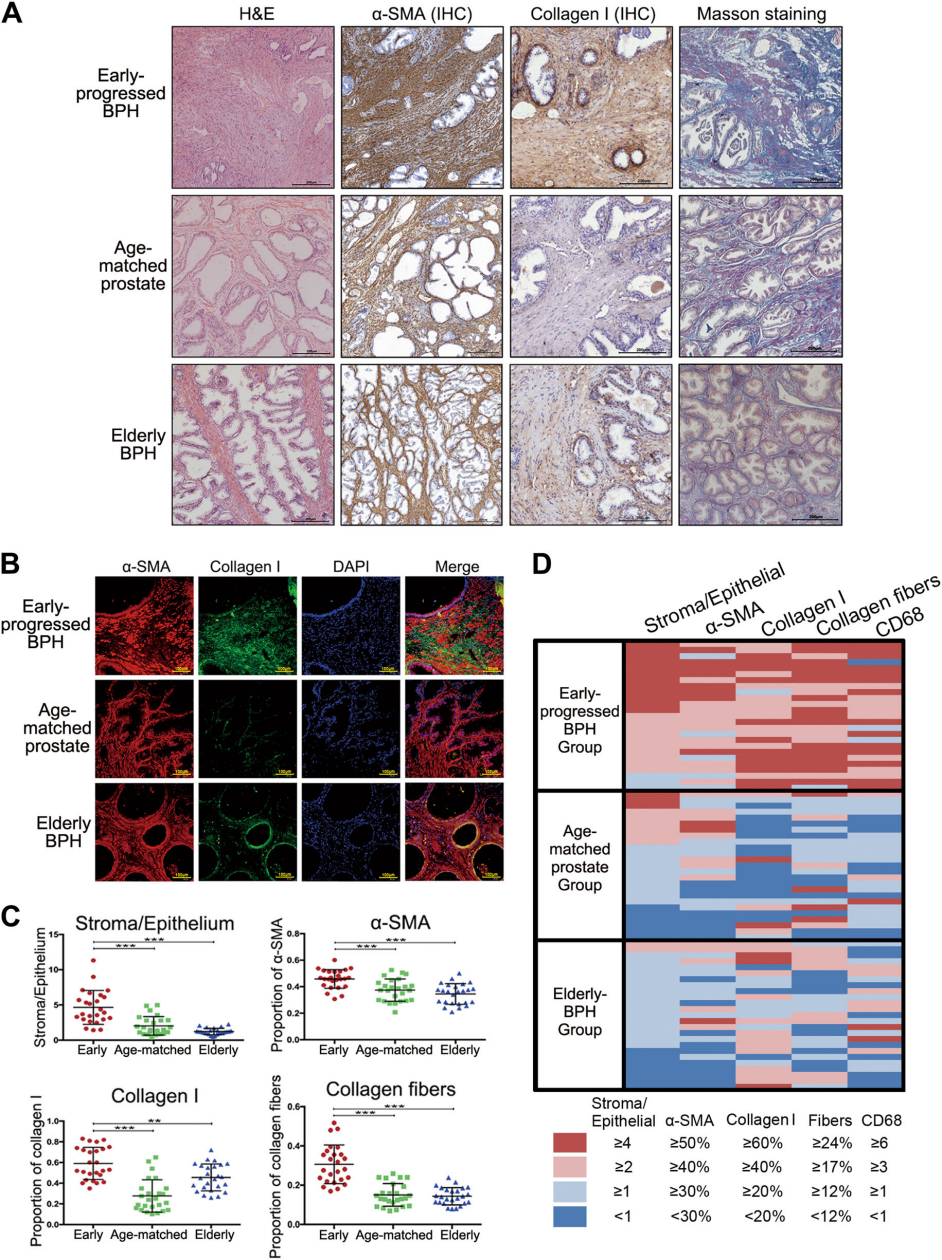
Early-progressed benign prostatic hyperplasia (BPH) is a stromal component-dominant lesion with excessive collagen deposition. **a** Representative haematoxylin and eosin (H&E), immunohistochemical and Masson’s trichrome- staining images showing α-SMA and collagen I expression in the early-progressed BPH, age-matched prostate and elderly BPH tissues. Masson’s trichrome-staining images show the smooth muscle cell cytoplasm in red and the extracellular collagen fibre in blue. Scale bar, 200 μm. **b** Representative immunofluorescence staining results showing α-SMA (red) and collagen I (green) expression. Scale bar, 100 μm. **c** Scatter plots showing the stroma-to-epithelium ratios, and the percent area densities of α-SMA, collagen I and collagen fibres in three analysed groups. *P < 0.05, **P < 0.01 and ***P < 0.001. **d** Heat map showing the distribution of and the relationship between the stroma-to-epithelium ratio, percent area densities of α-SMA, collagen I and fibres and CD68^+^ cell clusters in the three analysed groups

To investigate the histological characteristics of early-progressed BPH, we performed haematoxylin and eosin (H&E), immunohistochemical (IHC) and immunofluorescence (IF) staining to observe α-SMA and collagen I expression, and Masson’s trichrome staining to identify extracellular collagen fibres in all investigated samples (Fig. 1a, b). α-SMA, a myofibroblast and smooth muscle cell marker were shown to be diffusely expressed in the early-progressed BPH tissues. The α-SMA percent area densities were 0.46 ± 0.07, 0.37 ± 0.08 and 0.35 ± 0.08 (*P* < 0.001, compared to those in both the age-matched prostate and elderly BPH groups; Fig. 1c), while the stroma–epithelium ratios were 4.65 ± 2.41, 2.03 ± 1.32 and 1.23 ± 0.45 (*P* < 0.001, compared to those in the control groups; Fig. 1c) in the early-progressed BPH, age-matched prostate and elderly BPH groups, respectively. Additionally, collagen I, synthesised by the activated myofibroblasts, was shown to be widely expressed and deposited in the early-progressed BPH tissues compared to that in the age-matched prostate and elderly BPH groups (Fig. 1a, b; *P* < 0.001 and *P* < 0.01, respectively, Fig. 1c). Furthermore, Masson’s trichrome-staining results showed an increased level of collagen fibres in the stroma of the early-progressed BPH compared to those in the other two groups (*P* < 0.001 for both, Fig. 1a, c). In the heat map presented in Fig. 1d, the distribution and relationship between the stroma–epithelium ratios and the marker expression levels in three groups are presented.

### M2 macrophages selectively induce myofibroblast phenotypes in the early-progressed BPH fibroblasts

**Fig. 2:**
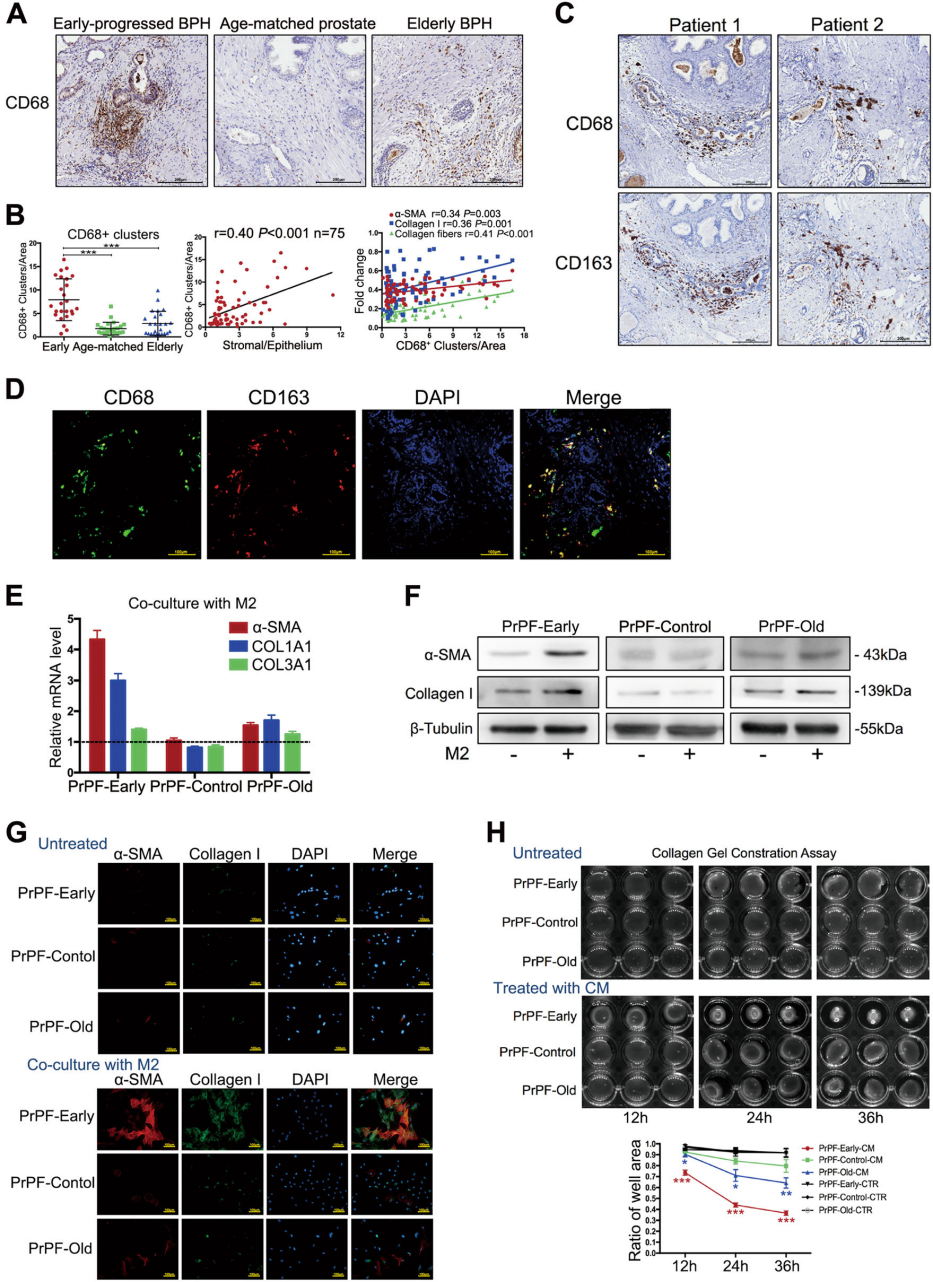
M2 macrophages selectively induce myofibroblast phenotype development in fibroblasts from the early-progressed BPH tissues. **a** Representative CD68 immunohistochemical staining images in the early-progressed BPH, age-matched prostate and elderly BPH tissues. Scale bar, 200 μm. **b** Scatter plots showing macrophage clusters in the three analysed groups. The number of macrophage clusters in prostate tissues correlated positively with the stroma-to-epithelium ratio, and percent area densities of α-SMA, collagen I and fibres. **c** Serial histological sections showing the early-progressed BPH tissues stained for CD68, a macrophage-specific marker, and CD163, an M2 macrophage marker. The distribution of CD68^+^ and CD163^+^ cells was almost identical. Scale bar, 200 μm. **d** Representative immunofluorescence (IF) staining images, showing CD68 (green) and CD163 (red) expression in the infiltrating macrophages in early-progressed BPH tissues. Scale bar, 100 μm. **e** Quantitative RT-PCR results showing α-SMA, COL1A1 and COL3A1 expression in primary prostate fibroblast (PrPF)-early, PrPF-control and PrPF-old cells co-cultured with THP-1-derived M2 macrophages. Data are shown as relative gene expression compared to that in the respective untreated fibroblasts. **f** Western blots showing α-SMA and collagen I protein expression in PrPF-early, PrPF-control and PrPF-old cells co-cultured with THP-1-derived M2 macrophages. **g** Representative IF staining results, showing the co-expression of α-SMA (red) and collagen I (green) following a 48-h co-culture of PrPF-early, PrPF-control and PrPF-old cells with THP-1-derived M2 macrophages. The respective untreated fibroblasts were used as controls. Scale bar, 100 μm. **h** Solidified collagen-gel shrinkage after the seeding of PrPF-early, PrPF-control and PrPF-old cells, untreated or treated with the conditioned medium. The respective untreated fibroblast samples were used as controls, and all assays were performed in triplicate. *P < 0.05, **P < 0.01, ***P < 0.001

Increased macrophage infiltration has been suggested to play an important role in BPH progression^11,12^ and pathological fibrosis development^13^. We found that the number of macrophage clusters expressing CD68, a macrophage-specific marker, significantly increased in the early-progressed BPH tissues (mean ± standard error of the mean (SEM): 7.9 ± 4.5), compared to those in the age-matched prostate (1.8 ± 1.4) and elderly BPH (2.9 ± 2.6) tissues (both *P* < 0.001; Fig. 2a, b). Additionally, the number of macrophage clusters in these patients was shown to correlate positively with the stroma–epithelium ratios (*r* = 0.40, *P* < 0.001) and the percent area densities of α-SMA (*r* = 0.34, *P* = 0.003), collagen I (*r* = 0.36, *P* = 0.001) and collagen fibres (*r* = 0.41, *P* < 0.001) (Fig. 2b). The distribution of and relationship between all these histological markers are shown in Fig. 1d.

To identify the macrophage subtype found in the early-progressed BPH tissues, we analysed the expression of CD68 and CD163 (M2 macrophage markers) in serial histological sections and observed a highly consistent distribution of CD68^+^ CD163^+^ clusters (Fig. 2c). IF staining further confirmed CD68 and CD163 co-expression in the infiltrating macrophages of the early-progressed BPH tissues (Fig. 2d), showing that the macrophages were indeed M2-type macrophages.

To investigate the potential roles of infiltrating M2 macrophages in the prostate stroma of the early-progressed BPH, we established an M2 macrophage and prostate fibroblast co-culture model. Fibroblasts from three different sources (PrPF-early, PrPF-control and PrPF-old) were used for these cultures. The expression of α-SMA and collagen I, encoded by COL1A1 gene, was increased in PrPF-early cells (Fig. 2e–g; Supplementary Figure S3A and S3B), compared with that in the other groups. Conditioned media (CM) samples were collected to perform collagen gel contraction assays, in order to verify if the addition of CM increases PrPF-early cell contraction in the collagen gels. After 36 h, the collagen gels were shown to shrink significantly compared to those containing the untreated early-progressed BPH fibroblasts (36.6% vs. 91.6% of the original area, respectively; *P* < 0.001; Fig. 2h). The average gels shrunk to 36.6%, 79.7% and 64.2% of the original gel area after a 36-h CM treatment on PrPF-early, PrPF-control and PrPF-old samples, respectively (Fig. 2h). In contrast, M2 macrophages did not affect α-SMA and collagen I mRNA and protein expression (Fig. 2e–g; Supplementary Figure S3A and S3B) or collagen-gel contraction when analysing the PrPF-control samples, and both effects were weak in the PrPF-old samples (Fig. 2h).

### TGFβ1 and IL4 are the potential mediators of M2 macrophage-induced myofibroblast phenotype

**Fig. 3:**
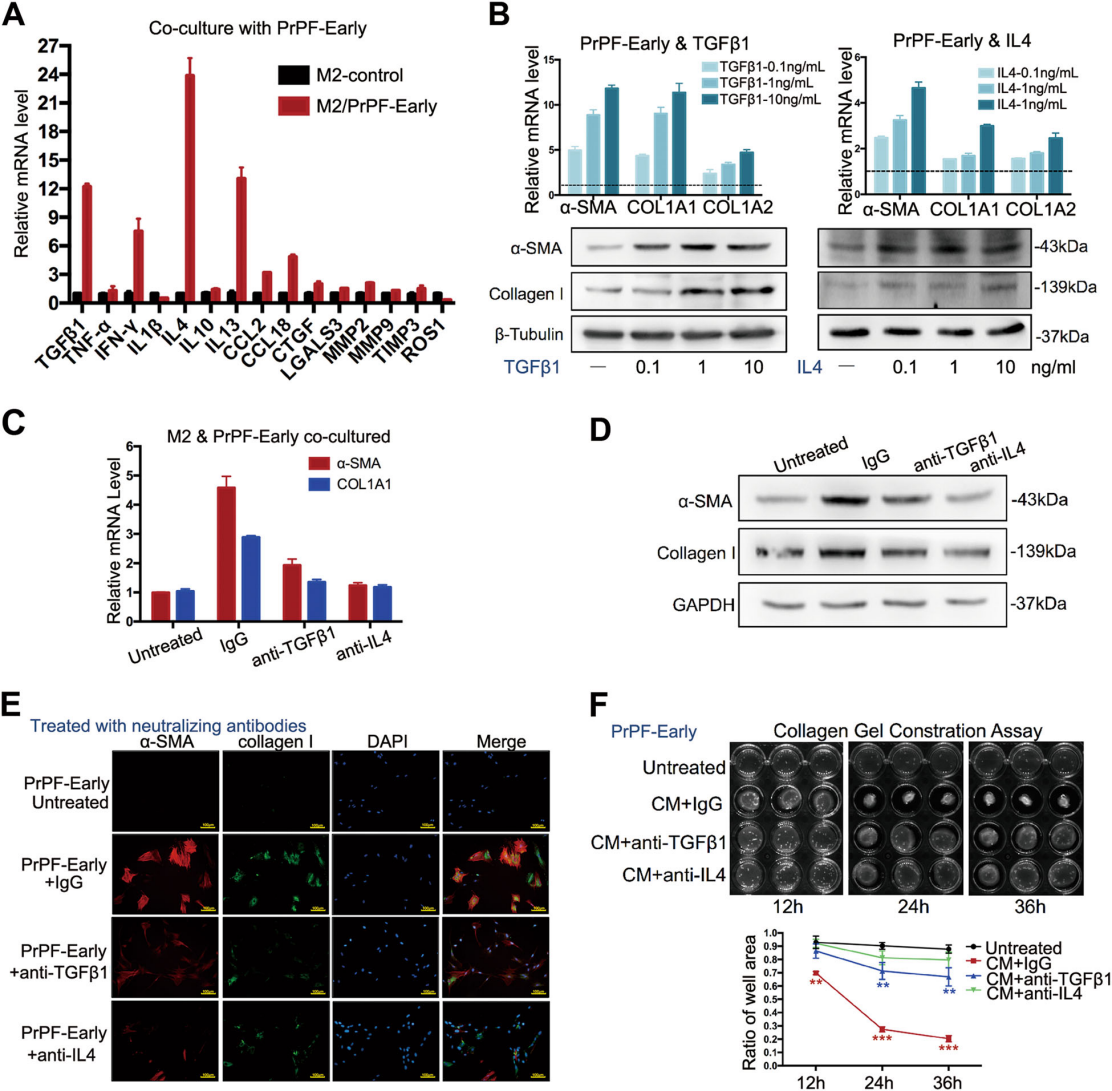
TGFβ1 and IL4 may mediate the development of M2 macrophage-induced myofibroblast phenotype. **a** Pro-fibrotic cytokine expression levels in the THP-1-derived M2 macrophages, co-cultured or not, with PrPF-early cells. **b** α-SMA, COL1A1 and COL3A1 expression, at mRNA and protein levels, following the exogenous addition of cytokines to PrPF-early samples compared to that in the untreated samples. The relative mRNA expression was compared with that in the untreated samples. **c**–**f** α-SMA and collagen I expression assessed using quantitative RT-PCR (**c**), western blotting (**d**), immunofluorescence staining (**e**) and contractility of PrPF (**f**) following the addition of anti-TGFβ1 (1 μg/ml), anti-IL4 (1 μg/ml) and IgG isotype (1 μg/ml) antibodies to the THP-1-derived M2 macrophage and PrPF-early cell co-cultures for 48 h, respectively. IgG isotype antibody was used as a control. Scale bar, 100 μm. All assays were performed in triplicate. *P < 0.05, **P < 0.01, ***P < 0.001

To identify the pro-fibrotic cytokines responsible for M2 macrophage-induced fibroblast-to-myofibroblast differentiation in the early-progressed BPH, we analysed the expression of cytokines potentially involved in the crosstalk between macrophages and fibroblasts. Of these, a considerable increase in TGFβ1, IL4, IFN-γ and IL13 expression in the THP-1-derived M2 macrophages was observed after their co-culture with PrPF-early cells, compared to those in the monocultured M2 macrophages (Fig. 3a). Furthermore, by adding increasing concentrations of the aforementioned cytokines to the PrPF-early cell culture, we determined that TGFβ1 and IL4 induce the expression of α-SMA and collagen I while IFN-γ and IL13 did not (Fig. 3b and Supplementary Figure S4).

To determine the effect of TGFβ1 and IL4 inhibition on the fibroblast gene expression and contractions, we treated the co-cultured M2-fibroblasts with anti-TGFβ1 and anti-IL4 antibodies (1 μg/ml). This treatment inhibited α-SMA and collagen I expression in PrPF-early cells, and was accompanied by a decrease in fibroblast contractility, compared to that in the isotype IgG control (Fig. 3c–f). The anti-IL4 antibody was shown to prevent the development of the myofibroblast phenotype more effectively than anti-TGFβ1 in the PrPF-early cells.

### IL4, but not TGFβ1, selectively induces myofibroblast phenotype in fibroblasts isolated from early-progressed BPH tissues

**Fig. 4:**
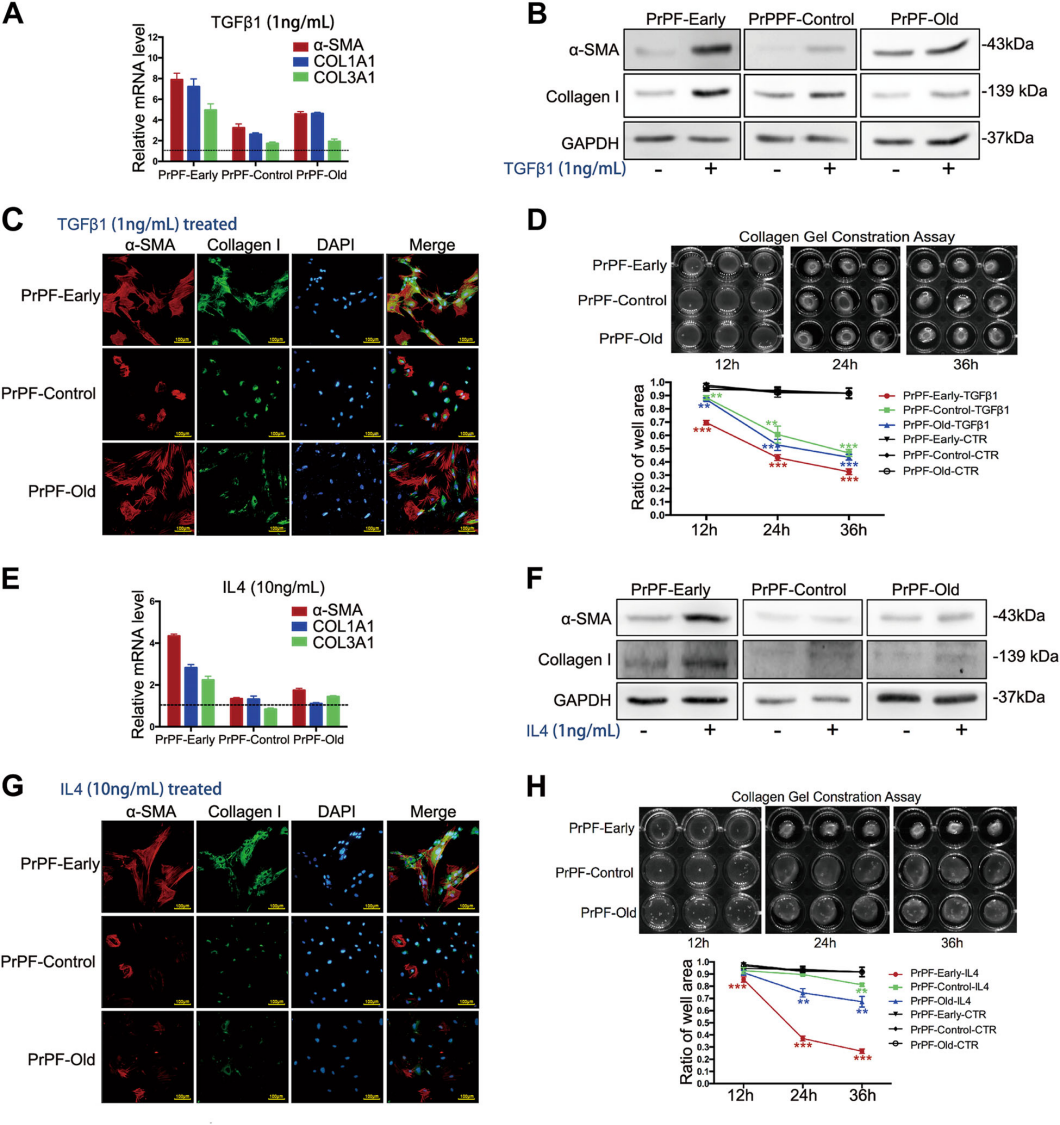
TGFβ1 and IL4 induce myofibroblast phenotype development in the primary prostate fibroblasts (PrPFs). **a** Quantitative RT-PCR results showing α-SMA, COL1A1 and COL3A1 expression in PrPF-early, PrPF-control and PrPF-old samples following a 48-h treatment with 1.0 ng/ml TGFβ1. Relative expressions compared to those in the untreated controls are presented. **b** Western blotting results showing α-SMA and collagen I expression in PrPF-early, PrPF-control and PrPF-old cells following the treatment with 1 ng/ml TGFβ1. **c** α-SMA (red) and collagen I (green) immunofluorescence (IF) staining of the PrPF-early, PrPF-control and PrPF-old cells following their treatment with 1 ng/ml TGFβ1. The respective untreated fibroblast-staining results are presented in Fig. 2g. Scale bar, 100 μm. **d** Cell contractility in all the analysed samples following 1 ng/ml TGFβ1 treatment compared to that in the untreated controls presented in Fig. 2h. **e** α-SMA, COL1A1 and COL3A1 mRNA expression levels in PrPF-early, PrPF-control and PrPF-old samples following the treatment with 10 ng/ml IL4 or in untreated fibroblasts. **f** Western blots showing α-SMA and collagen I expression in IL4-treated (10 ng/ml) PrPF-early, PrPF-control and PrPF-old cells. **g** α-SMA (red) and collagen I (green) IF-staining images following the treatment with 10 ng/ml IL4. The respective untreated fibroblasts were used as controls (Fig. 2g). Scale bar, 100 μm. **h** PrPF-early, PrPF-control and PrPF-old cell contractility following the treatment with 10 ng/ml IL4. The respective untreated fibroblasts were used as controls (Fig. 2h). All assays were performed in triplicate. *P < 0.05, **P < 0.01, ***P < 0.001

We further studied the pro-fibrotic effects of TGFβ1 and IL4 on the PrPF-early, PrPF-control and PrPF-old cells. Following the treatment with 1.0 ng/ml TGFβ1 for 48 h, the expression of α-SMA and collagen I increased in all three PrPF cell types, as demonstrated by qPCR, western blotting and IF (Fig. 4a–c). However, these effects were bigger in the PrPF-early samples. All analysed fibroblasts showed increased contractility in the collagen gels when treated with 1.0 ng/ml TGFβ1, and the gels shrunk to 32.6%, 46.8% and 43.5% of the original gel areas in the PrPF-early, PrPF-control and PrPF-old samples, respectively (Fig. 4d).

However, exogenous addition of 10 ng/ml IL4 induced the expression of α-SMA and collagen I only in the PrPF-early cells (Fig. 4e–g). In addition, collagen gel contraction assays demonstrated that the exposure of cells to IL4 for 36 h increased cell contraction, with the gel shrinking to 26.6% of the original area in the PrPF-early samples (Fig. 4h), and to 81.2% and 67.3% in the PrPF-control and PrPF-old samples, respectively. IL4 had either a weak or no effect on the myofibroblast differentiation and contractility in the PrPF-control and PrPF-old samples (Fig. 4e–h).

**Fig. 5:**
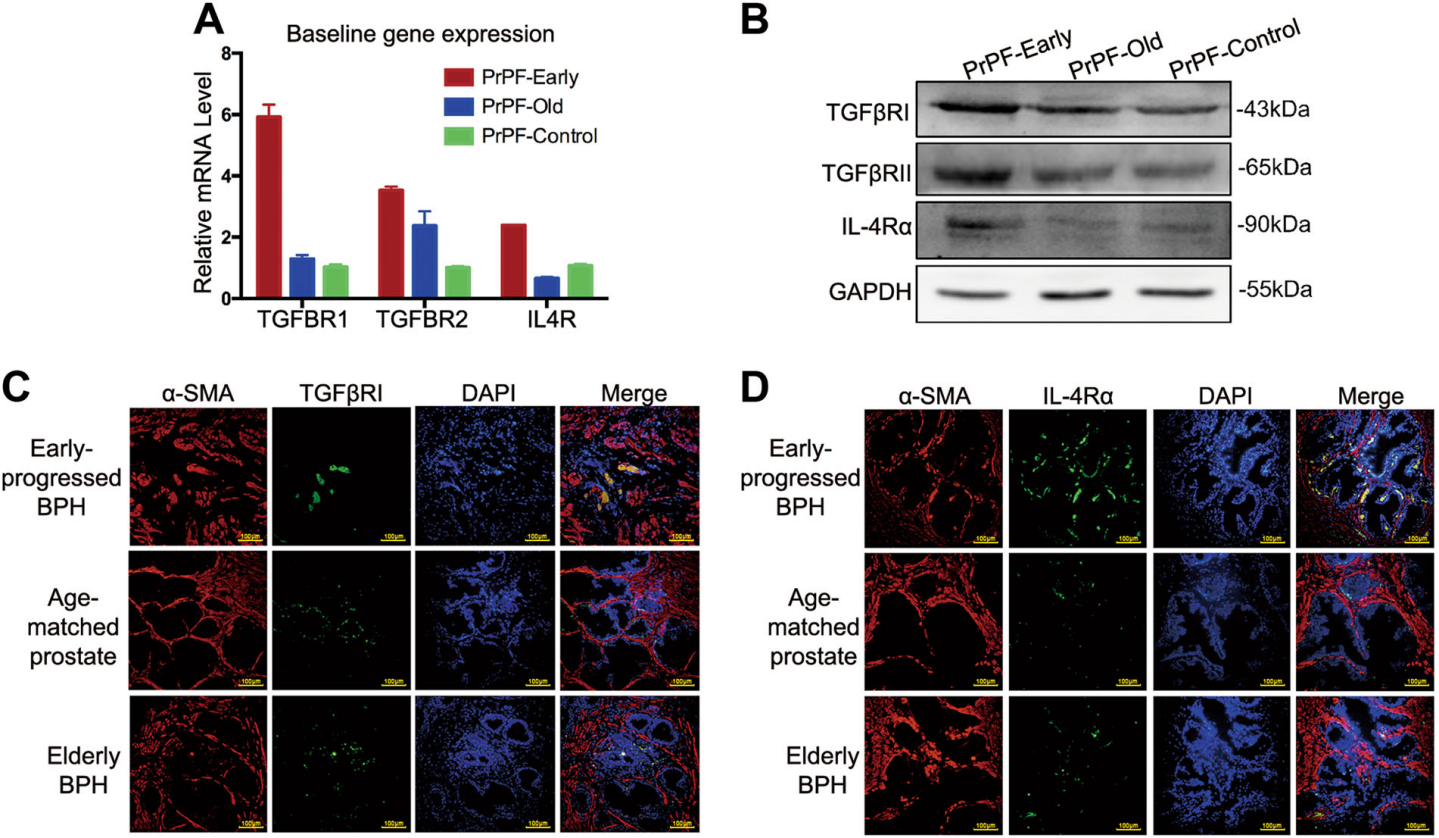
TGFβ1 and IL4 receptor expression in primary prostate fibroblasts (PrPFs) and prostate tissues obtained from the three analysed groups. **a**, **b** mRNA and protein expression levels of TGFBR1, TGFBR2 and IL4R in PrPF-early, PrPF-control and PrPF-old cells, respectively. The relative gene expression levels were normalised to the baseline expression in the PrPF-control samples. **c**, **d** Representative immunofluorescence staining images of the early-progressed BPH, age-matched prostate and elderly BPH tissues showing the co-expression of α-SMA and TGFβRI/IL4Rα in the fibroblasts. Scale bar, 100 μm

To understand the mechanisms underlying the observed effects, we investigated the expression of TGFβ1 and IL4 receptors in fibroblasts and prostate tissues. Our results demonstrated that the TGFβ receptors, TGFβRI and TGFβRII, were expressed by all fibroblasts, while the IL4 receptor, IL4Rα, was shown to be primarily expressed by PrPF-early cells (Fig. 5a, b). Furthermore, co-staining of α-SMA and TGFβRI or IL4Rα demonstrated an increase in the co-expression of α-SMA and the corresponding receptor in stromal cells of the early-progressed BPH tissues (Fig. 5c, d).

### M2 macrophage-mediated independent signalling pathways promote myofibroblast phenotype through TGFβ1 and IL4 activity

**Fig. 6:**
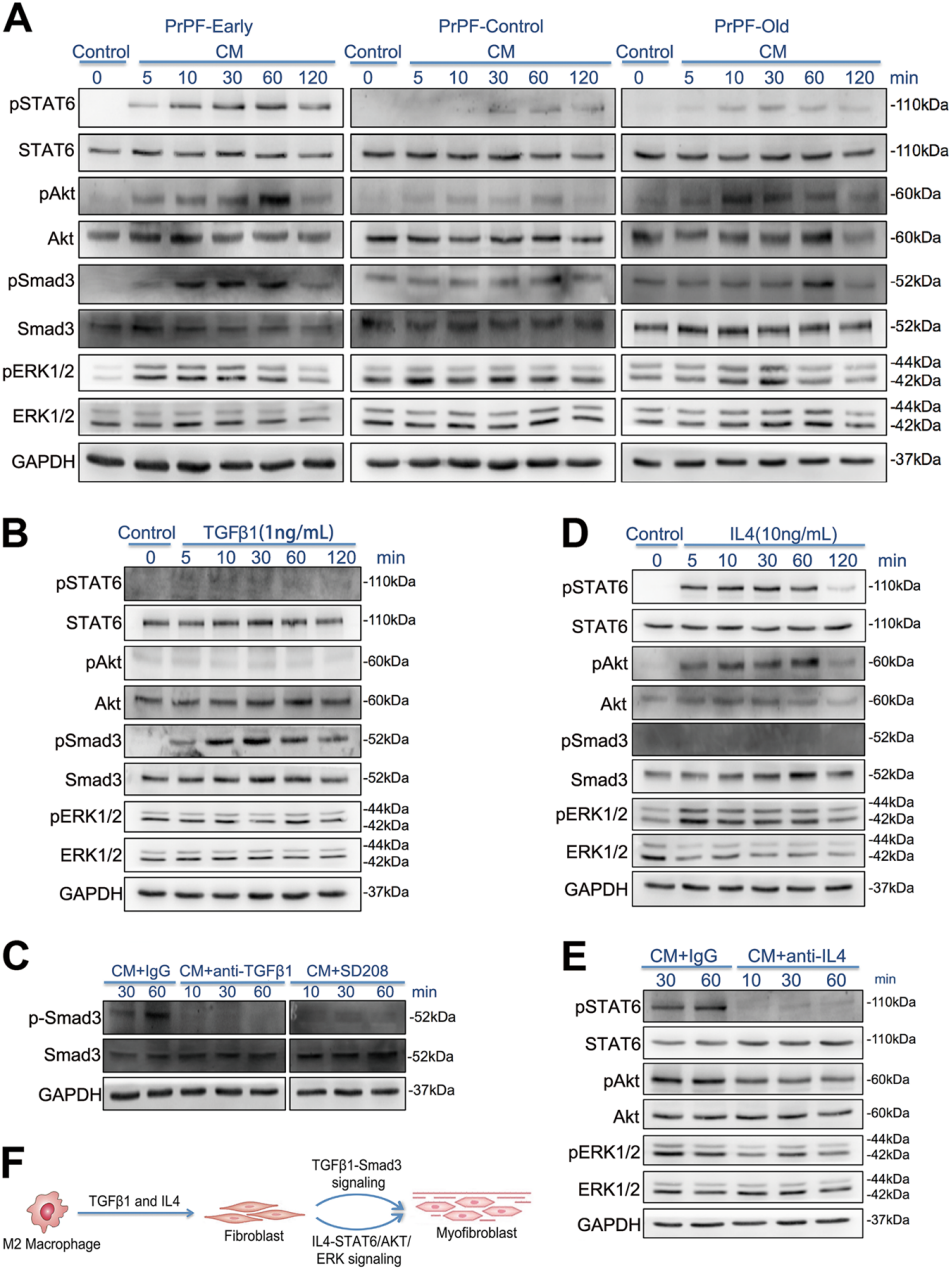
Mechanisms underlying M2 macrophage-mediated development of myofibroblast phenotype in different primary prostate fibroblasts (PrPFs). **a** SMAD3, STAT6, AKT and ERK phosphorylation levels in PrPF-early, PrPF-control and PrPF-old cells treated with the conditioned media (CMs) obtained from the THP-1-derived M2 macrophage–fibroblast co-cultures. **b** SMAD3, STAT6, AKT and ERK phosphorylation levels in PrPF-early, PrPF-control and PrPF-old cells treated with 1 ng/ml TGFβ1. **c** SMAD3 phosphorylation levels in PrPF-early cells following the treatment with CM pretreated for 3 h with 1 μg/ml anti-TGFβ1 antibody. Additionally, PrPF-early cells were pretreated with 1 μM SD208, a small-molecule ALK5 inhibitor of the TGFβ1R1/SMAD2/3 interaction, for 3 h. IgG isotype antibody (1 μg/ml) was used as a control. **d** JAK/STAT6, PI3K/AKT, SMAD3 and MAPK/ERK signalling following the treatment with 10 ng/ml IL4 in PrPF-early cells. **e** STAT6, AKT and ERK phosphorylation following the antibody-induced inhibition of IL4 for 3 h, prior to the treatment of PrPF-early cells with these media. CMs pretreated with 1 μg/ml IgG isotype antibody were used as controls. **f** Schematic illustration of the mechanisms underlying M2 macrophage-induced development of myofibroblast phenotype through the activation of TGFβ1 and IL4 signalling

Previous studies have demonstrated that TGFβ1- and IL4-induced fibrosis is associated with the activation of SMADs, JAK/STAT6, PI3K/AKT and MAPK/ERK signalling^14–19^. Therefore, we used the CMs from the co-cultures of M2 macrophages and PrPF cells to stimulate the corresponding fibroblasts and identify fibrosis-inducing signalling pathways. Our results demonstrated that the CM promotes SMAD3 phosphorylation, leading to a strong activation of STAT6, AKT and ERK signalling pathways in the PrPF-early cells; these changes are less pronounced in the other samples (Fig. 6a).

The treatment with 1 ng/ml TGFβ1 considerably induced SMAD3 phosphorylation in PrPF-early cells (Fig. 6b), and this effect was inhibited by treatment with an anti-TGFβ1 antibody (1 μg/ml) or the TGFβRI inhibitor SD208 (Fig. 6c). On the other hand, 10 ng/ml IL4 was shown to promote JAK/STAT6, PI3K/AKT and MAPK/ERK-mediated signalling (Fig. 6d), and the antibody-induced inhibition of IL4 activity resulted in a complete inhibition of STAT6 phosphorylation and a partial inhibition of AKT and ERK (Fig. 6e). Taken together, these data indicate that M2 macrophages promote fibroblast-to-myofibroblast differentiation in PrPF-early cells through TGFβ1-mediated TGFβ/SMAD3 signalling, and IL4-activated JAK/STAT6, PI3K/AKT and MAPK/ERK signalling pathways (Fig. 6f).

## Discussion

Although BPH is a slowly-progressive disease in elderly men, early-progressed BPH is a special type, with rapid clinical progression and severe LUTS, requiring surgery before the age of 50. Our findings demonstrate that early-progressed BPH is a fibroproliferative disease, and that the aberrant myofibroblast accumulation and collagen deposition are most likely responsible for BPH progression. Regardless of the initiating events, a key mediator of fibrotic tissue remodelling is the activation of extracellular matrix (ECM)-producing myofibroblasts, which express α-SMA and collagen I. The incorporation of α-SMA into stress fibres increases myofibroblast contractility, while collagens represent a prominent component of myofibroblast-produced ECM^20^. The persistent activity and resistance to apoptosis of myofibroblasts may induce ECM deposition in the prostate transitional zone tissues, causing tissue stiffness and low urethral compliance in men with LUTS^21^. Therefore, the differentiation of fibroblasts into myofibroblasts was suggested to be a key driver of the early-progressed BPH.

Furthermore, we showed that M2 macrophages induce myofibroblast phenotype and promote stromal fibrotic tissue remodelling in the early-progressed BPH. Previously, M2 macrophages were suggested to promote in vitro fibrogenic activities of fibroblasts by providing potent fibrogenic growth factors, including TGFβ1 and PDGFs^22^. Additionally, lung-specific overexpression of IL10 was shown to induce M2 macrophage activation and fibrocyte recruitment, driving the development of lung fibrosis^23^. Here, we confirmed that the increased expression of TGFβ1 and IL4 is essential for M2 macrophage-mediated myofibroblast phenoconversion in the early-progressed BPH tissues. We showed that TGFβ1 induces myofibroblast phenotype development, regardless of the origin of cells, while IL4 selectively induces myofibroblast phenotype only in prostate fibroblasts obtained from the patients with early-progressed BPH. We examined the effect of targeted cytokine inhibition in the macrophage–fibroblast co-culture system and found that an IL4-neutralising antibody has a stronger effect than an anti-TGFβ1 antibody. These results indicated that the inhibition of TGFβ1 is only partially responsible for the downregulation of the myofibroblast phenotypes. The IL4-neutralising antibody, however, could strikingly reverse the myofibroblast phenotype by blocking the pro-fibrotic effect of IL4 and activation of M2 macrophages. Therefore, M2 macrophage-derived IL4 may represent a more important pro-fibrotic cytokine than TGFβ1.

Using a macrophage–fibroblast co-culture model, we determined that M2 macrophages promote selective activation of the TGFβ/SMAD3 and IL4/STAT6/AKT/ERK signalling in the fibroblasts from the early-progressed BPH. Selective effects may be a consequence of different TGFβ1 and IL4 receptor levels in prostate fibroblasts of different origins. TGFβ1 initiates intracellular signalling by binding to cell-surface receptors, with TGFβRII activating TGFβRI, which then induces the phosphorylation of receptor-activated SMAD proteins, leading to pro-fibrotic effects^14,24^. On the other hand, IL4 binds to the IL4 receptor, which consists of the IL4Rα and the common γC chain^25,26^. Here, the expression of TGFβRI, TGFβRII and especially IL4Rα was shown to be increased in the early-progressed BPH-derived fibroblasts and tissues, which may partially explain the differential pro-fibrotic effects of M2 macrophages on prostate fibroblasts of different origins. Additionally, IL4Rα can be induced by cytokines such as IL4^27^, suggesting that the high expression levels of IL4Rα in the early-progressed BPH tissues may be due to an increase in M2 macrophage infiltration, which produces IL4 that acts in a paracrine manner, affecting the fibroblasts and inducing the expression of its receptor.

A better understanding of the histologic features and the underlying molecular pathways may offer a more cogent strategy aimed at decelerating the progression of prostate fibrosis and BPH treatment. According to previously held notions, the development of BPH-related symptoms and pharmacotherapeutic effects was related to the histological composition of the prostate tissues in patients with BPH. Pharmacotherapy is presently being targeted to induce epithelial atrophy of the prostate gland with 5α-reductase inhibitors and relaxing the prostate smooth muscle with alpha-blockers^28^. In our study, however, early-progressed BPH was found to be a fibroproliferative disease with excessive collagen deposition, which may account for the rapid progression of clinical symptoms and poor effectiveness of pharmacotherapy in most young patients with BPH. Therefore, the mechanisms that regulate aberrant myofibroblast accumulation and collagen deposition in BPH could be the potential targets for novel therapeutic approaches. Clinical studies showed that anti-IL4 agents may be used as therapies for the treatment of allergic rhinitis, asthma^29^ and many malignant tumours^30^. In addition, IL4 is a Th2-associated cytokine that alternatively activates M2 macrophages and stimulates the differentiation of fibroblasts into myofibroblasts, which show increased IL4Rα expression. Therefore, a selective targeting of the IL4/IL4Rα-mediated signalling pathway may be beneficial for the prevention of BPH progression.

This study, however, has several limitations. Previous findings had demonstrated that genetic background, hormonal alterations and metabolic profiles can lead to BPH progression^31–33^, but the underlying mechanisms are yet to be defined. We have not investigated the effect of these factors in the current study. We aimed to evaluate prostate tissue hormonal levels and identify gene expression and metabolomics profile using the fresh-frozen tissues from patients with early-progressed BPH. These investigations will help us clarify the aetiology of BPH progression in a more detailed and comprehensive manner.

## Materials and methods

### Patient specimens and ethics statement

Formalin-fixed paraffin-embedded (FFPE) prostate tissue blocks obtained from three groups of patients (25 cases in each) were included in this study: (1) prostate tissues of patients with early-progressed BPH, aged ≤50 years, obtained during the transurethral resection of the prostate (TURP); (2) age-matched prostate tissues, acquired from patients with bladder cancer, aged ≤50 years, who underwent radical cystectomy and prostatectomy and (3) prostate tissues of elderly patients with BPH, aged ≥75 years, obtained during TURP. The latter two represented the controls groups. Patients with prostate cancer and prostatitis, and those treated with alpha-adrenergic receptor antagonists or 5α-reductase inhibitors, were excluded. All retrospective clinical data analyses and prostate specimen collection were performed after obtaining informed consent from all patients and approval of the Peking University First Hospital Institutional Review Board. Patient information is included in Supplementary Table S1.

### Primary prostate fibroblast (PrPF) and M2 macrophage cultures

Three human primary fibroblast cultures, PrPF-early, PrPF-control and PrPF-old were obtained from the early-progressed BPH, age-matched prostate and elderly BPH tissues, respectively (Supplementary Figure S1A). Briefly, fresh prostate tissues were dissected into small fragments, and primary fibroblasts were isolated and cultured as described previously^34,35^. We performed several identification experiments to guarantee the purity of the primary fibroblasts (Supplementary Figure S1B and S1C). Fibroblasts were cultured in a DMEM:F12 1:1 (Thermo Fisher Scientific, MA, USA) mixture supplemented with 10% FBS (Thermo Fisher Scientific, MA, USA), 1% penicillin–streptomycin, 1 μg/ml EGF (PeproTech, NJ, USA) and 1 μg/ml bFGF (PeproTech, NJ, USA) at 37 °C in a humidified incubator with 5% CO_2_. After two generations, the resulting prostate fibroblasts were used in subsequent experiments.

Human acute monocytic leukaemia cells (THP-1, from American Type Culture Collections, VA, USA) were treated with 100 ng/ml PMA (PeproTech) for 3 days (d) until the formation of M0 macrophages, which were further differentiated for 5 d with 50 ng/ml IL4 (PeproTech) to obtain M2 macrophages (Supplementary Figure S2A)^36,37^. THP-1 monocytes became adherent, with a star-like phenotype, and the expression of several M2 macrophage marker genes was shown to be significantly increased (Supplementary Figure S2B). Additionally, human peripheral blood mononuclear cells (PBMCs) were isolated from the whole blood of patients with BPH, using Ficoll-Paque (Biochrom, Berlin, Germany) density gradient centrifugation. Human monocytes were isolated via flow cytometry with a CD14-FITC antibody (BD Biosciences, CA, USA), and treated with 1000 units/ml rh GM-CSF (PeproTech) for 7 d to obtain macrophages (Supplementary Figure S2C). The mRNA expression of M2 markers (Arg1, CCL18, CD163, CD206 and FIZZ1) was increased in the induced macrophages compared to that in human monocytes (Supplementary Figure S2D).

For the M2 macrophage–fibroblast co-culture system, fibroblasts of different origins were seeded into six-well plates, and M2 macrophages were added into transwells inserted in each well (pore size 0.4 μm, Corning, NY, USA). Three origins of fibroblasts, M2 macrophages and CM were harvested for subsequent experiments after 72 h of co-culture.

### RNA extraction and quantitative PCR

Total RNA was extracted using TRIzol reagent (Life Technologies, CA, USA) as previously described^38^. Purified RNA was quantified using a NanoDrop ND-1000 spectrophotometer (NanoDrop Technologies, DE, USA), and 1 μg of total RNA was reverse transcribed using a TransScript First-Strand cDNA Synthesis SuperMix (TransGen Biotech, Beijing, China). Quantitative PCR (qPCR) was performed using an ABI 7500 Real-time PCR system (Applied Biosystems, CA, USA) with GoTaq qPCR Master Mix (Promega, WI, USA) to determine the level of mRNA expression of target genes. The relative expression levels were normalised to that of GAPDH, were compared with the level of target gene expression in the baseline cell types or with the level of gene expression in the PrPF-control, which was assigned the value of 1 using 2^−ΔΔCT^ method. The primers were synthesised by Sangon Biotech (Shanghai, China; Supplementary Table S2).

### Western blots

Protein extraction and western blot assays were conducted as previously described^39^. The primary antibodies against α-SMA (ab7817, 1:300 dilution), collagen I (ab138492, 1:1000 dilution), GAPDH (ab8245, 1:5000 dilution), Smad3 (ab40854, 1:1000 dilution) and phospho-Smad3 (Ser 423 and Ser 425, ab52903, 1:1000 dilution) were purchased from Abcam (MA, USA); STAT6 (#9362, 1:1000 dilution), phospho-STAT6 (Tyr641, #9361, 1:1000 dilution), Akt (#9272, 1:1000 dilution), phospho-Akt (Ser473, #9271, 1:1000 dilution), ERK 1/2 (p44/42 MAPK, #4695, 1:1000 dilution) and phospho-Erk1/2 (Thr202/Tyr204, #4370, 1:1000 dilution) from Cell Signaling Technology (MA, USA) and IL4Rα (YT2337, 1:1000 dilution), TGFβ RI (YT4627, 1:1000 dilution) and TGFβ RII (YT4628, 1:1000 dilution) from ImmunoWay (TX, USA).

### H&E, Masson’s trichrome and IHC staining

FFPE prostate tissue blocks, obtained from the three groups of patients, were used for H&E and Masson’s trichrome staining, using standard procedures, and examined under a light microscope (Carl Zeiss, Göttingen, Germany); IHC staining was performed as previously described^40^. We first quantified the relative amounts of stromal components in the prostate tissue samples by using α-SMA (ab7817, 1:100 dilution), which stained smooth muscles and myofibroblasts in dark brown, leaving epithelial cells unstained. A total of ten fields at 40× magnification were examined for each histologic section, representing ~50% of the cross-sectional areas on one slide. Therefore, α-SMA-negative areas represented epithelial components, while the stromal area was calculated by subtracting the α-SMA-negative area from the total tissue area. Furthermore, the extent and intensity of collagen I (Abcam, ab138492, 1:1000 dilution) positive staining were evaluated using Image-Pro Plus 6.0 software; we calculated the percentage of collagen I-positive area and staining intensity of the stroma (weak staining, 0.5; moderate staining, 1 and strong staining, 1.5). The final staining extent score was determined by multiplying the percentages of collagen I-positive area with the intensity score. Additionally, we determined the number of CD68^+^ clusters by using the CD68 antibody (OriGene, MA, USA; UM800047, 1:200 dilution) to evaluate the degree of macrophage infiltration. A CD68^+^ cluster was defined as more than 60 CD68^+^ cells per field at 200× magnification. In this manner, the number of macrophage clusters per unit area of each prostate tissue section was determined.

### Immunofluorescence

Immunofluorescence (IF) staining of the prostate fibroblasts and FFPE sections was carried out as previously described^41^. The primary antibodies included mouse monoclonal α-SMA (Abcam, ab7817, 1:100 dilution), CD163 (OriGene, TA506380, 1:200 dilution) and EpCAM (Cell Signaling Technology, #2929, 1:800 dilution) antibodies, and the rabbit primary antibodies included collagen I (Abcam, ab138492, 1:800 dilution), CD68 (Proteintech, 25747-1-AP, 1:400 dilution), IL4R (ImmunoWay, YT2337, 1:400 dilution), TGFβ RI (ImmunoWay, YT4627, 1:400 dilution) and vimentin (Cell Signaling Technology, #5741, 1:100 dilution) antibodies. Fluorochrome-conjugated goat anti-mouse IgG (Alexa Fluor 488, Invitrogen, CA, USA; A10680, 1:400 dilution) and goat anti-rabbit IgG (Alexa Fluor 594, Invitrogen; A11012, 1:400 dilution) were used as secondary antibodies. DAPI Fluoromount-G (Southern Biotech, AL, USA) was used to identify cell nuclei, and fluorescence was detected with a Zeiss Axiophot photomicroscope (Carl Zeiss).

### Collagen gel contraction assay

Collagen contractility is a characteristic of functional myofibroblasts, and can be evaluated using a collagen gel contraction assay. The collagen gels were prepared on ice by mixing 0.2 ml of 3 mg/ml type I rat-tail collagen (Gibco, CA, USA) with 0.4 ml of RPMI-1640 medium (Corning); addition of 4 μL of 1 M NaOH produced a well-solidified gel with a neutral pH. Five-hundred microlitres of the mixture was immediately transferred to each well in a 24-well plate, and the gels were allowed to solidify at room temperature (20–25 °C) for 20 min. Next, 500 μL of monocultured media or co-cultured CM were gently added to each well. The plate was then gently swirled to ensure that the gel was entirely detached from the plate. An appropriate amount of cell (0.1–0.2 ml) suspension (1.5 × 10^5^ cells/ml) per well was seeded onto the solidified collagen gels. The 24-well plate was then placed in an incubator at 37 °C with humidified 5% CO_2_ air. The contraction of gels was observed, and the change in the diameter of the gels was recorded at several time points (12, 24 and 36 h) by using a digital camera at a fixed distance above the gels.

### Statistical analysis

Values are presented as the mean ± SEM from at least three independent experiments. Differences in mean values between two groups were analysed with the two-tailed Student’s *t* test. IHC data were analysed through the Spearman’s rank correlation test. For all statistical tests, *P* values <0.05 were considered statistically significant. All statistical analyses were performed using the SPSS 22.0 software (IBM, NY, USA).

## Electronic supplementary material

Supplementary Figures

Supplementary Tables

Supplementary figure legends
