# Supplementary material for: M2 macrophage-mediated interleukin-4 signalling induces myofibroblast phenotype during the progression of benign prostatic hyperplasia
Source: Cell Death Dis. 2018 Jul 9;9(7):755. doi: 10.1038/s41419-018-0744-1 (PMC6037751; doi:10.1038/s41419-018-0744-1)
Supplement: Supplementary file 1 — Supplementary Figures [file 41419_2018_744_MOESM1_ESM.docx]

**Supplementary Figures**


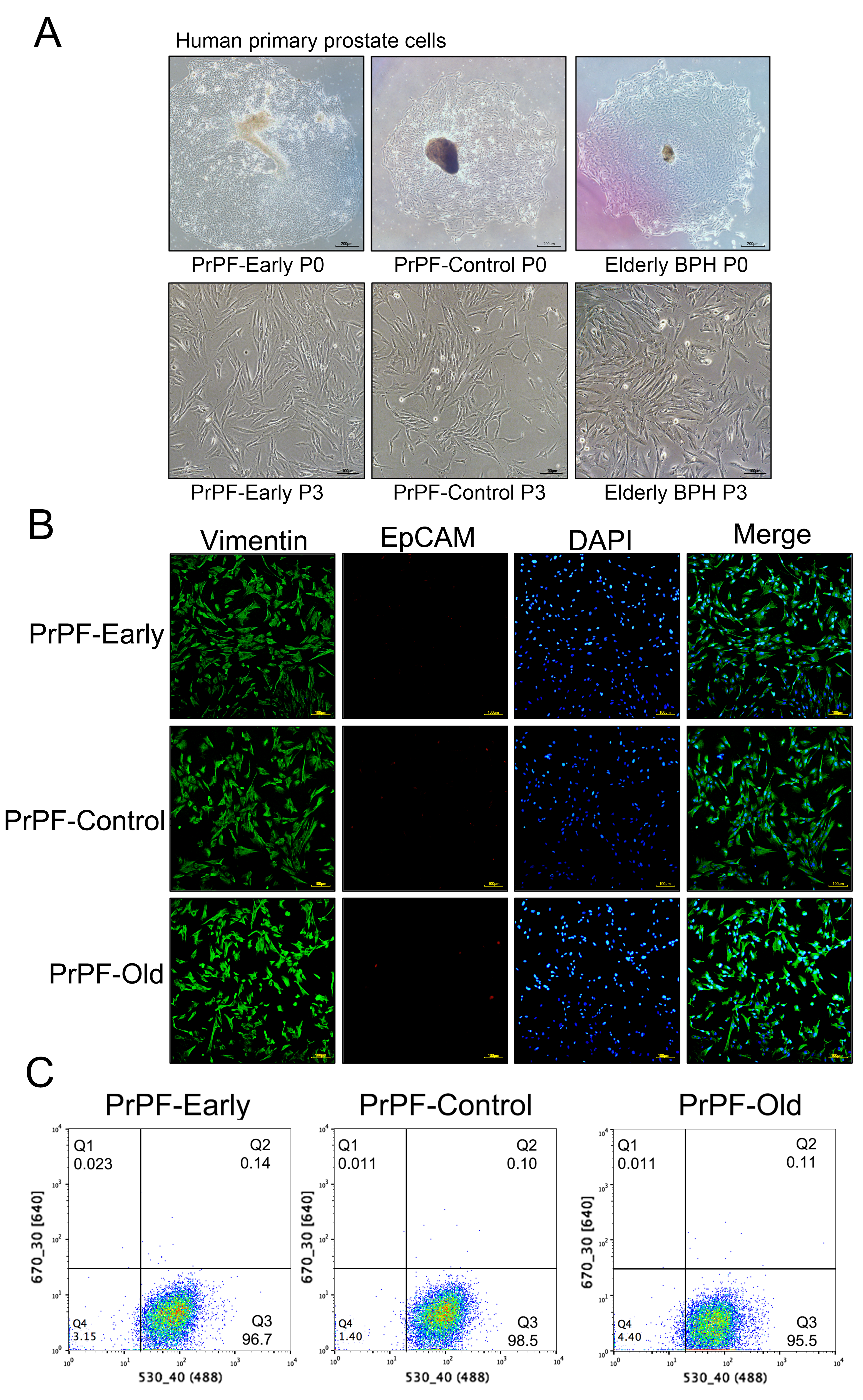


Supplementary Figure S1. Establishment and identification of human primary prostate fibroblasts (PrPFs). (A) Human primary prostate fibroblasts, designated as PrPF-Early, PrPF-Control, and PrPF-Old were isolated from the prostate tissues of early-onset BPH, age-matched prostate tissues, and elderly BPH tissues, respectively. (B) Immunofluorescence staining, showing the expression of fibroblast marker vimentin (green) and the epithelial cell marker EpCAM (red) in the PrPF-Early, -Control, and -Old samples. Magnification, 100×；scale bar 100 μm. (C) Flow cytometric results, showing the proportions of fibroblasts in PrPF-Early, -Control, and -Old samples. Representative flow cytometry scatter plots showing the proportion of vimentin-FITC positive and EpCAM-APC negative (fibroblasts) cells are presented.


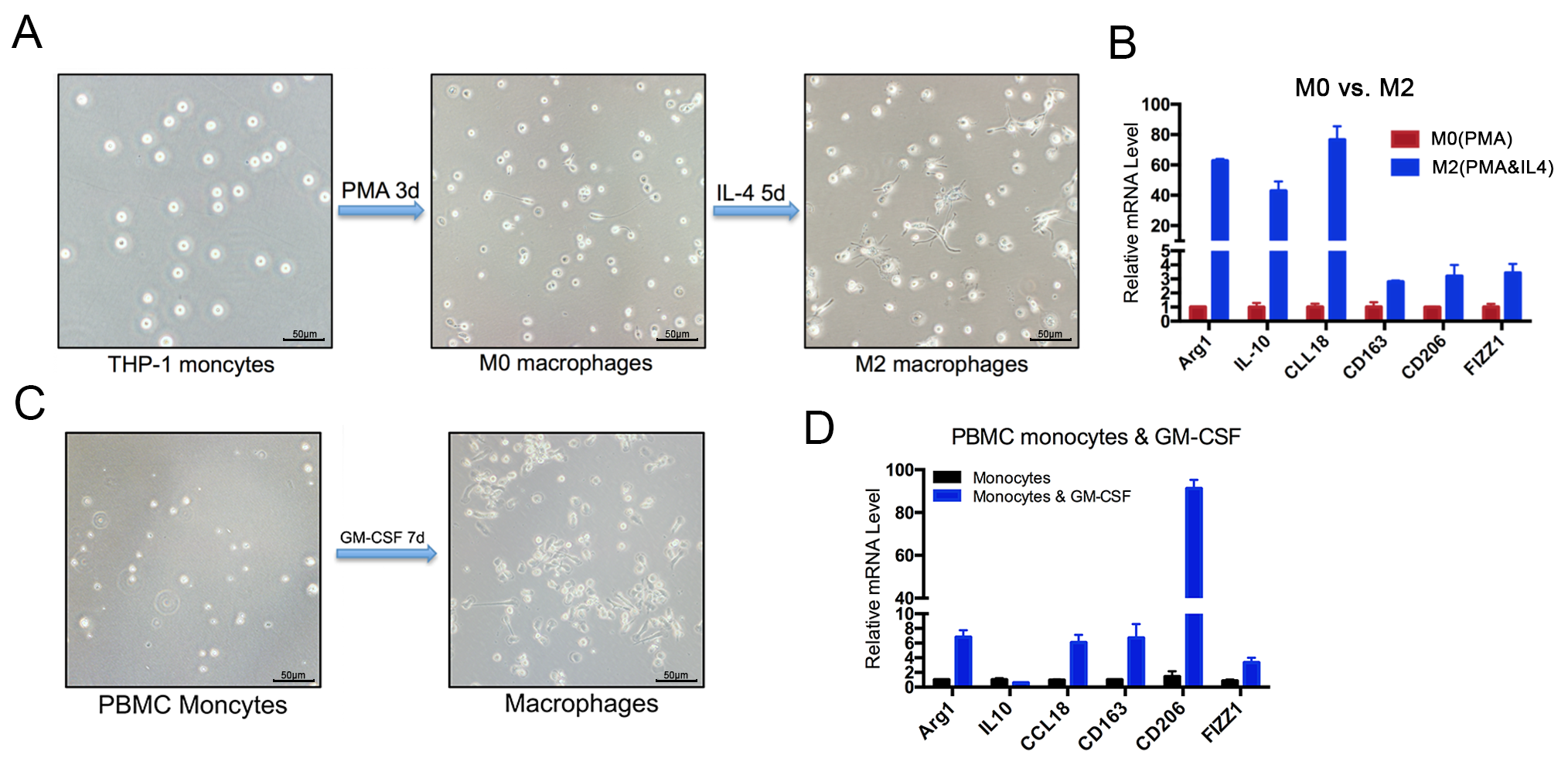
Supplementary Figure S2. Establishment and identification of human acute monocytic leukemia cell line (THP-1) and primary moncytes derived M2 macrophages. (A) THP-1 was stimulated by phorbol 12-myristate 13-acetate (PMA) and interleukin-4 (IL4) sequentially to obtain M2 macrophages. Scale bar 50 μm. (B) Quantitative RT-PCR analysis for specific M2 markers (Arg1, IL10, CCL18, CD163, CD206 and FIZZ1) between THP-1 induced M2 and M0 macrophages. (C) Human peripheral blood monocytes were treated with 1000 units/ml rh GM-CSF for 7 d to obtain macrophages. (D) The expressions of M2 markers in the induced macrophages examined by qRT-PCR.


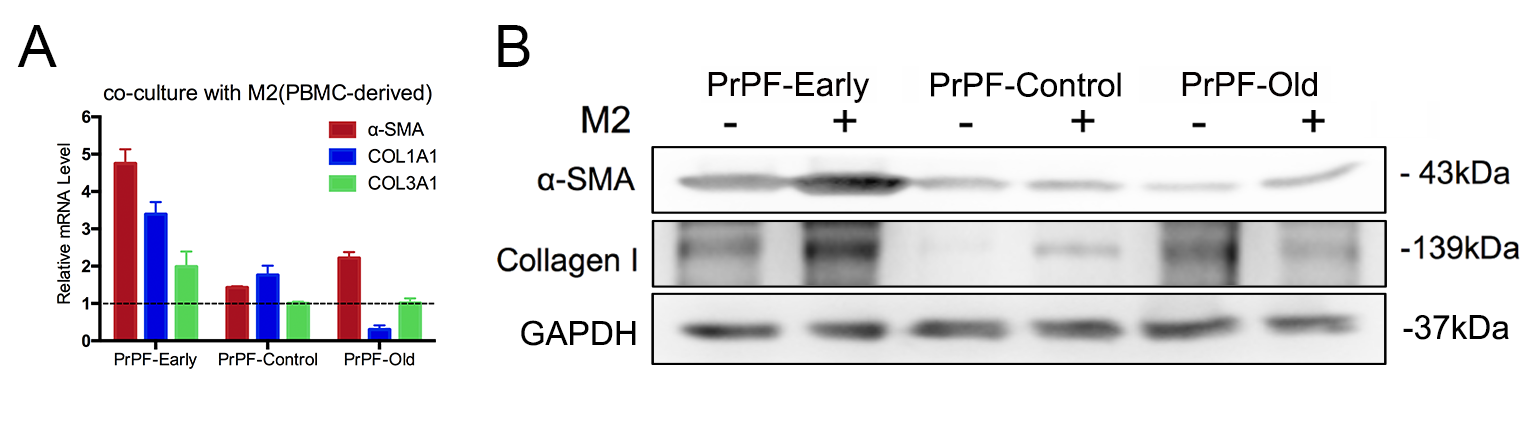


Supplementary Figure S3. Human peripheral blood monocytes-derived M2 macrophages selectively induce myofibroblast phenotype in fibroblasts from the early-progressed BPH tissues. (A) Quantitative RT-PCR results, showing α-SMA, COL1A1 and COL3A1 expression in the primary prostate fibroblast (PrPF)-Early, -Control, and -Old cells co-cultured with peripheral blood monocyte-derived M2 macrophages. Data are presented as relative gene expression compared with that in the respective untreated fibroblasts. (B) Western blots results, showing α-SMA and collagen I expression in the PrPF-Early, -Control, and -Old cells co-cultured with peripheral blood monocyte-derived M2 macrophages.


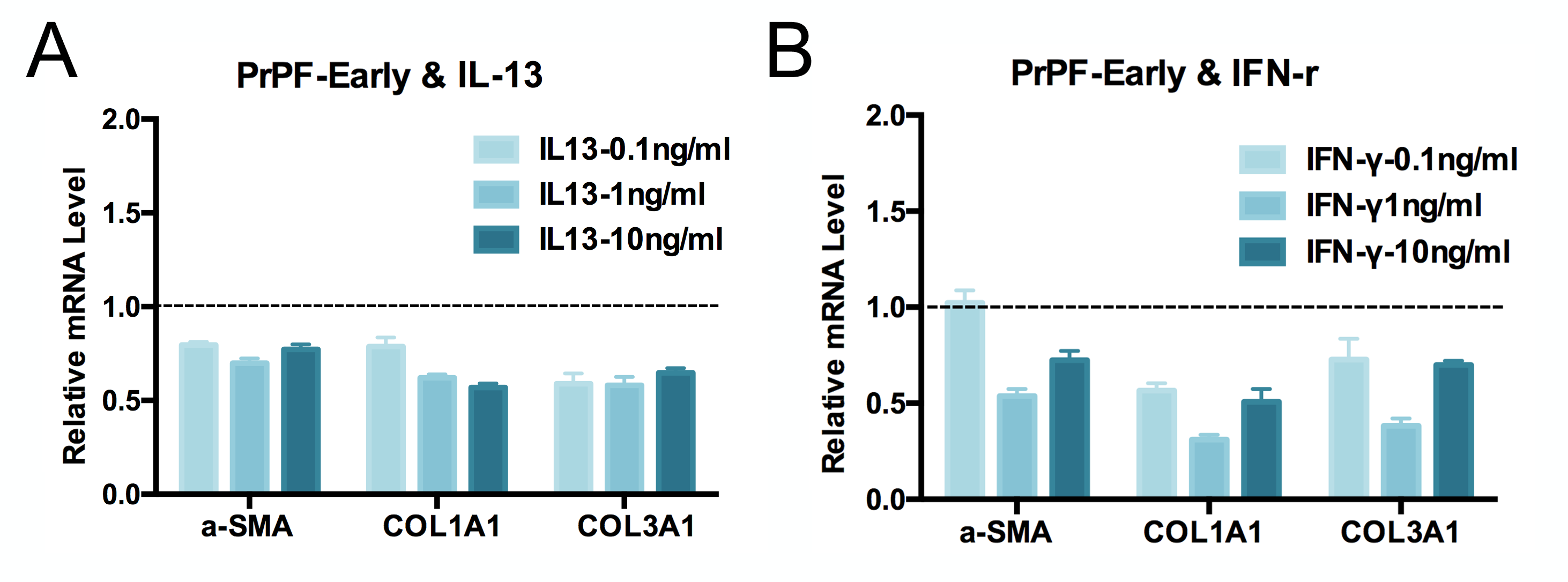


Supplementary Figure S4. IL13 and IFN-γ could not induce the myofibroblast phenotype in PrPF-Early. (A, B) With the exogenous addition of gradient concentrations (0.1,1,10 ng/ml) IL13 and IFN-γ to PrPF-Early for 48 h, the expressions of α-SMA, COL1A1 and COL3A1 were not increased when assess by quantitative RT-PCR.
