# Supplementary material for: M2 macrophage-mediated interleukin-4 signalling induces myofibroblast phenotype during the progression of benign prostatic hyperplasia
Source: Cell Death Dis. 2018 Jul 9;9(7):755. doi: 10.1038/s41419-018-0744-1 (PMC6037751; doi:10.1038/s41419-018-0744-1)
Supplement: Supplementary file 2 — Supplementary Tables [file 41419_2018_744_MOESM2_ESM.docx]

**Supplementary Tables**
